# Chemotherapy Enhances Cross-Presentation of Nuclear Tumor Antigens

**DOI:** 10.1371/journal.pone.0107894

**Published:** 2014-09-22

**Authors:** Chidozie C. Anyaegbu, Richard A. Lake, Kathy Heel, Bruce W. Robinson, Scott A. Fisher

**Affiliations:** 1 National Centre for Asbestos Related Diseases (NCARD), QEII Medical Centre, Nedlands, Western Australia; 2 School of Medicine and Pharmacology, University of Western Australia, QEII Medical Centre, Nedlands, Western Australia; 3 School of Pathology and Laboratory Medicine, University of Western Australia, QEII Medical Centre, Nedlands, Western Australia; Saint Louis University School of Medicine, United States of America

## Abstract

Cross-presentation of tumor antigen is essential for efficient priming of naïve CD8^+^ T lymphocytes and induction of effective anti-tumor immunity. We hypothesized that the subcellular location of a tumor antigen could affect the efficiency of cross-presentation, and hence the outcome of anti-tumor responses to that antigen. We compared cross-presentation of a nominal antigen expressed in the nuclear, secretory, or cytoplasmic compartments of B16 melanoma tumors. All tumors expressed similar levels of the antigen. The antigen was cross-presented from all compartments but when the concentration was low, nuclear antigen was less efficiently cross-presented than antigen from other cellular locations. The efficiency of cross-presentation of the nuclear antigen was improved following chemotherapy-induced tumor cell apoptosis and this correlated with an increase in the proportion of effector CTL. These data demonstrate that chemotherapy improves nuclear tumor antigen cross-presentation and could be important for anti-cancer immunotherapies that target nuclear antigens.

## Introduction

Recognition of tumor antigen by specific T cells is a necessary prerequisite for the induction of effective anti-tumor immune responses [1]. This is initiated by *cross-presentation*, a phenomenon where professional antigen presenting cells (APC) such as dendritic cells (DC) capture, process, and present exogenous antigens through the class I pathway [2]. Cross priming of naïve CD8 T cells by professional APC invokes a program leading to tumor specific-cytotoxic T lymphocytes (CTL) which proliferate and traffic to the tumor site where they ultimately attack and destroy tumor cells [3]. The efficiency of cross-priming has been shown to be influenced by the level of APC activation and maturation status [4], as well as the properties of cross-presented antigen itself. Factors such as antigen dose [5], type (modified self-antigen or mutated tumor neoantigen) [6], source (whether the antigen was derived from live or apoptotic cells) [7], [8], location of the antigenic determinant (epitope) within the tumor protein [9], and subcellular location within tumor cells [10], [11] can affect cross-priming efficiency. With respect to location, while some studies have shown that the cytoplasm is the main source of antigen, they typically compared the secretory, cell-membrane, and cytoplasmic compartments, but did not assess the contribution of nuclear antigen. Many of the tumor-associated antigen (TAAs) currently targeted in anti-tumor immunotherapy, such as survivin, MAGE-A10, and WT1, are predominantly expressed in the nucleus [12]–[14]. Moreover, several clinical studies have correlated the nuclear localization of different TAAs with poor clinical outcome in a variety of cancer types [15]–[20]. Therefore, it is important to understand the relative availability for cross-presentation of antigens in the nuclear compartment compared to other cellular compartments. We hypothesized that nuclear localization of an antigen would limit cross-presentation and hence cross priming of naïve CD8 T cells because APCs would not be able to access nuclear antigen as easily as antigen from other cellular locations. Furthermore, since apoptosis is known to cause nuclear degradation [21], [22] and extracellular release of nuclear contents [23], we reasoned that apoptosis-inducing chemotherapy would improve the efficiency of cross-presentation of nuclear antigens.

To address these questions, we developed a murine tumor model of cross presentation in which MHC mismatch between the tumor cell and model antigen precludes direct antigen presentation. We engineered H2-K^b^ B16 tumors to differentially express a model tumor antigen (influenza HA H2-K^d^ restricted CL4 epitope expressed in frame with EGFP) in the secretory, cytoplasmic, or nuclear compartments, and compared their potential to induce proliferation of H-2K^d^ restricted CL4-specific CD8^+^ T cells *in vivo* as a measure of antigen cross presentation. We then investigated the capacity of the tumor apoptosis-inducing chemotherapy agent, gemcitabine [24], to improve cross-presentation. Here, we show that nuclear antigen is not cross-presented as efficiently as its cytoplasmic and secreted counterparts, and that gemcitabine induced tumor cell apoptosis could reverses this, boosting nuclear antigen cross-presentation to equivalent levels *in vivo*, which correlated with an increase in the proportion of effector CL4-specific CTL.

## Materials and Methods

### Mice

Female, C57BL/6 (H-2^b^) x BALB/c (H-2^d^) F_1_ mice (H-2^bxd^), and Clone 4 (CL4) TCR transgenic mice, whose CD8^+^ T cells express TCRs that specifically recognize the H-2^d^ restricted ‘CL4’ epitope (IYSTVASSL; residues 518–526) of influenza haemagglutinin (HA) protein [25], were obtained from the Animal Resource Centre (Canning Vale, Perth, Australia). All mice were used at seven to ten weeks of age, and maintained under standard conditions (M-block Animal Facility, Queen Elizabeth II Medical Centre, The University of Western Australia; UWA, Perth, Australia). Animal experiments were approved by The University of Western Australia Animal Ethics Committee (RA/3/100/1016), and conducted in accordance with the Australian National Health and Medical Research Council's Code of Practice for the Use of Animals for Scientific Purposes.

### Tumor cell line and subcutaneous transplantation

B16.F10 (H-2^b^) melanoma tumor cell line (ATCC, Manassas, VA; CRL-6475) was used in all experiments. Tumor cells were maintained in RPMI-1640 (Invitrogen Life Technologies, Mulgrave, Australia) supplemented with 10% Fetal Calf Serum (FCS; Invitrogen Life Technologies), 50 µg/mL gentamicin (David Bull Labs, Kewdale, Australia), 60 µg/mL penicillin (CSL, Melbourne, Australia) 20 mM HEPES (Sigma Aldrich, Sydney, Australia) and 0.05 mM 2-mercaptoethanol (pH 7.2; Merck, Kilsyth, Australia). 5×10^5^ viable B16 cells in 100 µL PBS were inoculated subcutaneously (s.c.) into the lower right flank of recipient F_1_ mice. Tumor size was measured across two dimensions at least three times weekly with microcalipers. Mice were euthanized via Penthrane inhalation (Abbot Laboratories, USA) and death confirmed by cervical dislocation when tumors reached 100 mm^2^ as per animal ethics approval.

### Generation of gene constructs targeting model antigen to distinct subcellular compartments

The secretory gene construct (sec-EGFP-CL4) was generated by attaching the H-2K^b^ -derived endoplasmic reticulum (ER) signal peptide (MVPCTLLLLLAAALAPTQTRAV) to the amino-terminus of enhanced Green Fluorescent Protein (EGFP). A truncated version of the ER signal peptide (underlined sequence) was used to generate the cytoplasmic construct (cyto-EGFP-CL4), while the Simian Virus 40 T-antigen (SV40 T-Ag) nuclear localisation signal (MPKKKRKV), was used to generate the nuclear construct (nuc-EGFP-CL4). The following amino acid sequence QVGIYSTVASSLSELEKD was fused to the carboxyl-terminus of EGFP in all three constructs. This sequence encodes the CL4 epitope (underlined; the MHC Class I restricted region of Influenza haemagglutinin protein) preceded, and followed by, short spacer sequences to ensure that antigen presentation required cytosolic processing. All gene constructs were generated using PCR primers. Resulting PCR products were cloned into pCR4Blunt-TOPO (Zero Blunt PCR Cloning Kit; Invitrogen Life Technologies), and subcloned into pLenti6/V5-GW.blasticidin lentiviral vector, using BamH1/Xho1 cohesive ligation, for introduction into B16 cells. Sequence integrity of all clones was confirmed by DNA sequencing.

### Stable lentiviral transduction of B16 tumor cells with gene constructs

To generate lentivirus for integrating gene constructs into B16 target cells, 293FT lentiviral producer cells (Invitrogen Life Technologies) were co-transfected, using Lipofectamine™ 2000 transfection reagent (Invitrogen Life Technologies), with pHIV-PV-VSVG lentiviral packaging plasmid and pLenti6/V5-GW.blasticidin expression vector containing sec-EGFP-CL4, cyto-EGFP-CL4, or nuc-EGFP-CL4 gene constructs. Transfected 293FT cells were cultured in DMEM (Invitrogen Life Technologies) containing FCS (10%), L-glutamine (2 mM), non-essential amino acids (NEAA, 0.1 mM), penicillin (100 U/ml), and streptomycin (100 mg/ml). Lentivirus-containing supernatant was harvested after a 72-hour incubation, and added neat to a fresh culture of B16 cells. Stably transduced B16 cell were generated using blasticidin antibiotic selection (10 µg/mL in RPMI-1640, Invitrogen Life Technologies, for 10–12 days). Stable EGFP positive cells were sorted to a purity of >98% using BD Influx™ cell sorter (Becton Dickinson, San Jose, USA), and clonal populations expressing EGFP-CL4 protein in secretory (B16.Sec), cytoplasmic (B16.Cyto), or nuclear (B16.Nuc) subcellular compartments isolated by limiting dilution assay.

### Preparation of whole cell lysates and secretory fractions for EGFP ELISA assay

Whole cell lysates were extracted from each cell line by resuspending 5×10^6^ tumor cells in 500 µL of radio immuno-precipitation assay (RIPA) lysis buffer (150 mM sodium chloride; 1.0% Triton X-100; 0.1% sodium dodecyl sulphate; 50 mM Tris, pH 8.0; 1/100 dilution Protease Inhibitor Cocktail (P8340, Sigma Aldrich) and 0.5% sodium deoxycholate) and incubating for 30 minutes at 4°C. Cell debris was removed by centrifugation (500×g for 10 mins) and supernatants containing whole cell extracts gently transferred to a fresh tube. To generate secretory fractions, each cell line was seeded at 1×10^6^ cells/10 cm cell-culture dish. Following a 24-hour incubation, supernatant containing secretory fraction was harvested and concentrated using the Amicon Ultra-15 centrifugal 10 kDa filter device (Merck Millipore, Massachusetts, USA). Protein concentrations were determined by Bradford assay. EGFP-CL4 levels in cell lysates and fractions were then quantified using GFP ELISA assay kit (Cell Biolabs Inc. San Diego, USA), as per manufacturer's instructions.

### Confocal microscopy

EGFP expressing B16 cells were cultured on glass coverslips and fixed with 4% paraformaldehyde. Cell nuclei were counterstained with Hoechst 33342 nuclear envelope marker (Invitrogen Life Technologies), and confocal microscopy performed using a Nikon A1Si spectral detector confocal system (Nikon, Melville, USA). Imaging was performed with a 60× oil immersion objective lens. For each cell line, at least 10 single plane images of representative cells (optical slice  = 0.125 µm) were recorded. Images were analysed using the NIS-C Elements software (Nikon).

### Acquisition and analysis of ImageStream imaging flow cytometry data

The ImageStreamX imaging flow cytometer (Amnis, Seattle, USA) was used to confirm the differential expression of EGFP-CL4 antigen in respective B16 tumor cell lines. Briefly, each cell line was counter-stained with Hoechst 33342 nuclear marker and 10,000 cells acquired for analysis. Cell populations were hierarchically gated for single cells that expressed both EGFP and Hoechst 33342 as previously described [26]. Following acquisition, the relationship between EGFP and nuclear images was analysed using the “Similarity” feature of the IDEAS software (Amnis) [26]. The similarity score provides a measure of the degree of nuclear localisation of EGFP by comparing a log-transformed Pearson's correlation coefficient between the pixel values of two image pairs. Cells with a high similarity score exhibit strong correlation between images (i.e. EGFP is predominantly nuclear located), while cells with a low similarity score exhibit no correlation between images (i.e. EGFP is predominantly located within the cytoplasm).

### MTT assay

The metabolic activity (sensitivity) of B16 tumors after exposure to gemcitabine (Gem) was determined using the MTT [3-(4,5-dimethylthiazo-2-yl)-2,5-diphenyltetrazolium bromide] colorimetric assay (Sigma-Aldrich), according to manufacturer's protocol. Briefly, 24 h after seeding tumor cells to 96-well flat-bottomed tissue culture plates (1×10^3^ cells/well), serially diluted concentrations of Gem were added to wells. Following incubation (37°C in 5% CO_2_) for a further 48 h, 50 µL MTT solution (0.1 mg/well) was added and plates incubated for a further 4 hrs. Resulting formazan crystals were solubilized using dimethyl sulfoxide (DMSO, 100 µL/well; Sigma-Aldrich), and absorbance measured at 570 nm (Victor™ plate reader, PerkinElmer, Massachusetts, USA). All experiments were performed in triplicate.

### Carboxyfluorescein diacetate succinimidyl ester (CFSE)-based in vivo T cell proliferation assay

The CFSE-based T cell proliferation assay, originally described by Lyons and Parish [27], was used as an indirect measure of antigen cross-presentation. We used the mixing strategy of inoculating F_1_ mice with B16 parental cells mixed with B16.Nuc, B16.Cyto, or B16.Sec in different proportions, because a high CL4 antigen dose could saturate the proliferation of CL4-TCR transgenic T cells, and this has the potential to mask differences in cross-presentation. Homogenized single cell suspensions from spleens and lymph nodes [28] of naïve CL4 mice were labelled with CFSE (Molecular Probes, Invitrogen Life Technologies) as described previously [29]. Tumor-bearing F_1_ mice were then intravenously (i.v.) injected with CFSE-labelled lymphoid CL4 cells (1×10^7^ cells/mouse in 100 µL PBS). Seventy-two hours after adoptive transfer, F_1_ mice were culled and single cell suspensions prepared from their tumor draining LN (tdLN). Samples were labelled with CD8α-ef780-APC (clone 53-6.7, eBioscience, San Diego, USA) monoclonal antibody (mAb) and acquired on a FACS Canto II flow cytometer (Becton Dickinson, San Jose, USA). Cells were first gated on live lymphocytes before gating on CFSE^+^ CD8^+^ cells. Five hundred thousand events were collected and analysed using FlowJo software (TreeStar, Ashland, USA).

### Chemotherapy

Tumor-bearing F_1_ mice were intraperitoneally (i.p.) injected with a single dose of the apoptosis-inducing chemotherapeutic deoxycytidine analogue, gemcitabine hydrochloride (GEM. Sandoz. Pyrmont, Australia) at 240 µg/g body weight, in 100 µL of saline at indicated timepoints. Mice were monitored daily for potential weight loss and other toxicities.

### Statistical analysis

Data comparing differences between groups were assessed using the Student t test. Differences between growth curves were compared using ANOVA. Differences were considered significant when the P value was <0.05. Statistical analysis was conducted using GraphPad Prism 4.0 Software (San Diego, USA).

## Results

### Characterization of B16 tumors expressing a model tumor antigen in distinct cellular locations

**Figure 1:**
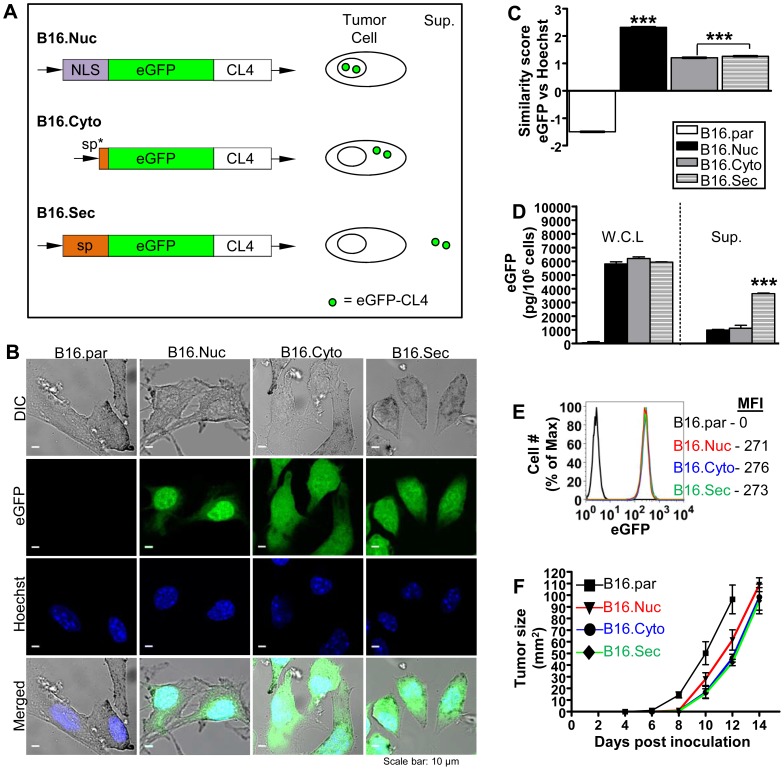
Schematic illustration and characterization of B16 tumor cells expressing differentially localized antigen. (A) Schematic depiction of gene constructs used to generate B16 parental (B16.par) tumors expressing EGFP-CL4 recombinant protein in secretory (B16.Sec), nuclear (B16.Nuc), or cytoplasmic (B16.Cyto) cellular compartments. (B) Confocal fluorescence microscopy demonstrated targeted expression of EGFP-CL4 in distinct subcellular compartments within respective tumors (magnification, ×60). (C) Quantitative imaging flow cytometry confirmed confocal microscopy observations. (D) EGFP ELISA was used to compare the amount of EGFP present in whole cell lysates (W.C.L) and supernatants (Sup.) of respective tumors. A standard curve of recombinant EGFP protein was used to calculate the amount of EGFP. A representative experiment is shown out of two independent ELISAs in triplicates ± SEM. (E) Relative intensity and stability of EGFP expression was determined by live-cell flow cytometry. Mean fluorescent intensities (MFI) are shown in figure. (F) *In vivo* growth pattern of respective tumors after s.c. inoculation in F1 mice. Data are means of 10 mice from two independent experiments. ***p<0.001. One-way ANOVA, followed by a Bonferroni post-test.

To study the effect of cellular localization on tumor-specific CD8^+^ T cell responses, we modified B16 tumor cells to express the MHC class I K^d^ restricted immunodominant epitope of the influenza virus HA protein, CL4, fused with EGFP, as a quantifiable model tumor antigen in nuclear (B16.Nuc), secretory (B16.Sec), or cytoplasmic (B16.Cyto) compartments (Fig. 1A). The dense nuclear expression of EGFP-CL4 in B16.Nuc, but diffuse cytoplasmic expression in B16.Cyto and B16.Sec tumors, demonstrated that antigen was successfully targeted to the intended cellular locations (Fig. 1B). These data were quantitated by imaging flow cytometry, a technique that combines the statistical power of flow cytometry with the spatial resolution of confocal microscopy. Based on the IDEAS algorithm [26], where similarity scores above 2 and below 1 indicate a high, or low degree of EGFP/nuclear co-localisation respectively, B16.Nuc had the highest EGFP/nuclear co-localisation with a mean similarity score of 2.317 (Fig. 1C). Due to the low error associated with analysing large numbers of cells (>10,000 per sample), we detected a statistically significant EGFP/nuclear co-localization in B16.Sec relative to B16.Cyto (Fig. 1C). As expected, B16.Sec secreted the highest concentration of EGFP-CL4 protein into culture supernatant (Fig. 1D).

Importantly, all three tumor cell lines expressed a similar steady-state whole cell level of EGFP-CL4 protein at approximately 6000 pg per 10^6^ cells, or 3.33×10^−12^ nmol of EGFP-CL4 per cell (Fig. 1D). These results were confirmed by the similar live-cell EGFP fluorescence intensities of respective tumors (Fig. 1E). This also confirmed stability of EGFP-CL4 fusion protein as the fluorescence intensities remained unchanged in long-term culture, without additional antibiotic selection, for over 50 passages (Fig. 1E). Although B16 parental tumor showed a slightly faster *in vivo* outgrowth, subsequent comparative studies confirmed that all three antigen-bearing tumors displayed a similar *in vivo* growth pattern (Fig. 1F). Taken together, these data demonstrate that B16.Nuc, B16.Cyto, and B16.Sec tumors targeted antigen to their respective cellular locations, and expressed similar levels of EGFP-CL4 fusion protein as well as *in vivo* growth pattern.

### Nuclear localized tumor antigen is not cross-presented as efficiently as cytoplasmic and secretory antigen

**Figure 2:**
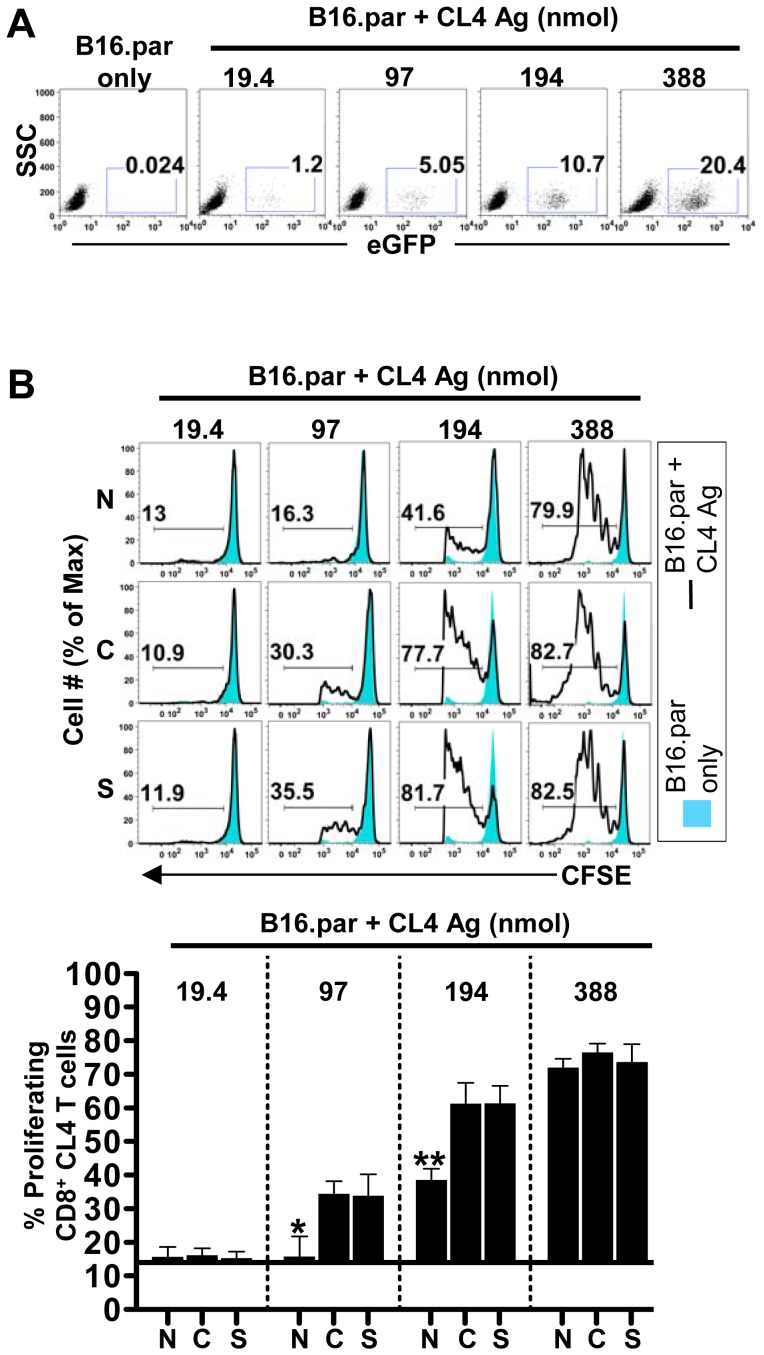
Nuclear localized tumor antigen was not cross-presented as efficiently as cytoplasmic and secretory counterparts. (A) The EGFP-CL4 antigen expression profile of B16 parental cells (B16 par.) or, B16 par. mixed with 1 (19.4), 5 (97), 10 (194), or 20% (388 nmol) of B16.Nuc (N), B16.Cyto (C), or B16.Sec (S) CL4 antigen-containing cells, was confirmed on the day of tumor inoculation by flow cytometry. For simplicity, Figure 2A only shows the profile of B16.Nuc mixtures, as they were not different to B16.Cyto or B16.Sec (B) CFSE-labelled CL4-specific CD8+ T cells were intravenously injected into F1 mice bearing 8-day-old subcutaneous tumors, and the proliferation of these T cells in the tumour draining lymph node examined on day 11 post-tumor inoculation by flow cytometry. Data are representative of two independent experiments, each involving five mice per group. Bars begin at the Mean ± SEM baseline proliferation for B16 parental cells.

Having generated the B16 tumors with differentially localized antigen, we proceeded to assess the impact of cellular antigen location on cross-presentation. Because our B16 tumors (H-2^b^) lacked the relevant restriction element (H-2^d^) for recognition by the CL4 TCR, we could rule out direct presentation of the antigen by the tumors [33] or by MHC-peptide exchange; a phenomenon described as “cross dressing” [20]. Thus in tumor-bearing C57BL/6 x BALB/C F_1_ mice (H-2^bxd^), the proliferation of adoptively transferred H-2^d^ restricted CL4-TCR transgenic CD8 T cells could only be induced when F1 host APCs cross-present CL4 antigen. In order to better define the threshold level of antigen that we previously reported to be required for cross-presentation [30], [31] and determine whether this threshold level differed depending on the cellular location of antigen, we inoculated F_1_ mice with B16 parental cells that had been mixed with either B16.Nuc, B16.Cyto, or B16.Sec tumor cells in different proportions, diluting the concentration of antigen to the doses shown in Fig. 2A. These experiments demonstrated that nuclear antigen required a twofold higher threshold concentration for cross-presentation relative to the cytoplasmic and secretory antigen; 194 nmol versus 97 nmol respectively (Fig. 2B).

### Gemcitabine induces apoptosis-mediated cell death of B16 tumor cells

**Figure 3:**
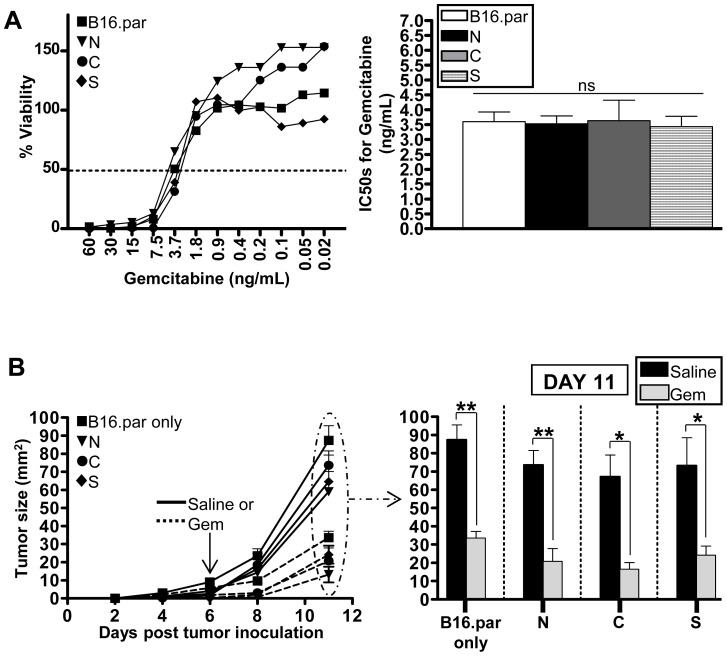
The apoptosis-inducing agent gemcitabine has a direct effect on B16 tumors in vitro and in vivo. (A) The relative in vitro sensitivity of B16 parental, B16.Nuc, B16.Cyto, and B16.Sec tumor cells to gemcitabine was assessed by MTT assay, after a 48 hr incubation with drug. With relevent concentrations of gemcitabine that kill 50% of respective tumors shown. (B) *In vivo* growth of B16 parental only, and B16 parental mixed with 10% (194 nmols) of B16.Sec-, Cyto-, or Nuc-CL4 antigen-containing tumors, after subcutaneous inoculation of F1 mice on day 0, and intraperitoneal administration of a single dose of gemcitabine (240 µg/g) or saline on day 6. Bar graph shows tumor sizes on day 11. For simplicity, Figure 3B only shows data from the 10% antigen concentration experiment as this was similar to that of 1 and 5%. Mean ± SEM. Six mice per group from two independent experiments. One-way ANOVA followed by a Bonferroni post test against all groups.

Since our data suggested that nuclear antigens are relatively inefficiently cross presented compared to other subcellular compartments, we hypothesised that this situation could be altered by gemcitabine, an immunogenic chemotherapy agent that we previously reported to increase the availability of antigen for cross-presentation by inducing apoptotic tumor cell death [24]. Importantly, B16 parental, B16.Nuc, B16.Cyto, and B16.Sec tumors were equally sensitive to gemcitabine with an IC50 between 3 and 4 ng/ml as determined by MTT assay (Fig. 3A). This sensitivity was also observed *in vivo*, as a single i.p. injection of gemcitabine (240 µg/g) significantly retarded the outgrowth of all B16 tumors compared to saline treated mice (Fig. 3B). Thus, gemcitabine induces similar levels of apoptosis-mediated cell death on all of the B16 tumors in this model.

### Gemcitabine improves cross-presentation efficiency of nuclear antigen in a dose dependent manner

**Figure 4:**
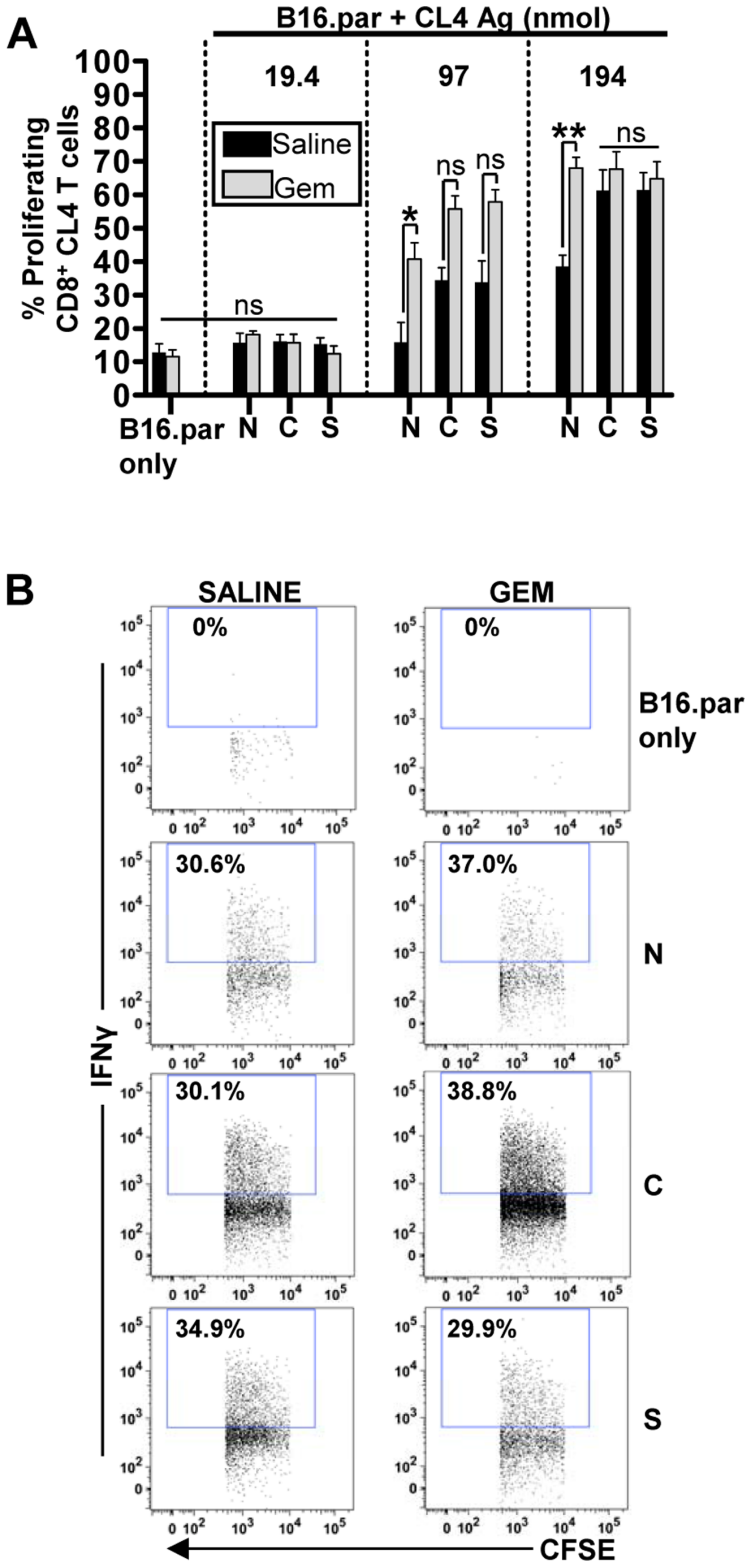
Gemcitabine improves the cross-presentation efficiency of nuclear localized antigen. F1 mice were inoculated on day 0 with B16 parental only (B16 only), or B16 parental mixed with 1 (19.4), 5 (97), or 10% (194 nmol), B16.Nuc (N), B16.Cyto (C), or B16.Sec (S) CL4 antigen-containing tumors, and a single dose of gemcitabine or saline administered on day 6. CFSE-labelled CL4 T cells were transferred on day 8, and the (A) proliferation and (B) Interferon gamma expression of CL4-specific CD8^+^ T cells in tumour draining lymph nodes of mice assessed on day 11. Mean ± SEM. Six mice per group from two independent experiments. One-way ANOVA followed by a Bonferroni post test against all groups. ns  =  not significant, *p<0.05, ***p<0.001.

After establishing the comparable sensitivity of B16 tumors to gemcitabine-mediated apoptosis, we proceeded to test whether gemcitabine could augment the cross-presentation efficiency of nuclear localized antigen and enhance cross presentation to a level similar to that seen with cytoplasmic and secreted tumor antigens. As all three tumors were similar in (i) their antigen content (Fig. 1E), (ii) were from the same parental line, (iii) grew at similar rates *in vivo* and (iv) exhibited comparable degrees of apoptosis-induced cell death, we can reasonably assume a similar amount of tumor debris following chemotherapy. Therefore, to determine if there was any change in the cross presentation of nuclear antigens following gemcitabine induced immunogenic cell death, F_1_ mice were inoculated with B16 parental cells mixed with either no antigen, or 19.4, 97, or 194 nmol of CL4 antigen-containing B16.Nuc, B16.Cyto, or B16.Sec tumor cells. Tumor bearing mice were treated with gemcitabine or saline and the proliferation of adoptively transferred CL4-specific CD8^+^ T cells in tumor draining LNs of mice assessed. Compared to saline, gemcitabine doubled the cross-presentation efficiency of nuclear antigen to levels comparable to the cytoplasmic and secretory antigen, both of which demonstrated only marginal increases relative to saline treated controls, thus abrogating the difference in cross-presentation efficiency between antigens in different cellular locations (Fig. 4A). Consequently, the threshold amount of antigen required for nuclear localized antigen to be cross-presented was lowered two-fold from 194 (with saline) to 97 nmol (Fig. 4A). Interestingly, while gemcitabine doubled the cross-presentation efficiency of nuclear antigen, the proportion of CL4-specific CD8 T cells expressing IFNγ remained unchanged (Fig. 4B).

## Discussion

Cross presentation is essential for the generation of effective anti-tumor immunity [3]. Tumor-specific CTL, with the capacity to destroy tumor cells, are only generated following effective cross-priming of naïve CD8 T cells by professional APC. To evaluate the efficiency of this process for different tumor compartments, we developed a murine model of tumor antigen cross-presentation, in which tumor cells differentially expressed a model antigen in either the secretory, cytoplasmic or nuclear compartments and compared their ability to induce proliferation of antigen-specific CD8 T cells as a read out of cross-presentation. We observed a significant difference in EGFP/nuclear localisation between secretory and cytoplasmic located tumor antigen. This is consistent with expression in the secretory pathway of secreted tumor antigen as secreted proteins show more nuclear co-localization than cytoplasmic proteins due to their transport through the endoplasmic reticulum, which borders the nuclear envelope [32], [33]. Despite this, all tumors expressed similar levels of tumor antigen in their respective cellular compartment and grew at comparable rates *in vivo*, indicating we had developed a robust model suitable for comparing the efficacy of nuclear antigen cross-presentation versus other compartments.

### Nuclear antigen is not cross-presented as efficiently as cytoplasmic or secreted antigen, but is restored following treatment with gemcitabine

Throughout this study, all tumor antigens were efficiently cross-presented when antigen concentration was sufficiently high. However, when lower tumor antigen concentrations were studied, we observed that nuclear tumor antigen was not as efficiently cross-presented, requiring at least a two-fold higher concentration relative to secreted or cytoplasmic tumor antigen to achieve similar level of CD8 T cell proliferation. We concluded that the reduced efficiency of cross-presentation was likely due to the distinct nuclear localization of tumor antigen, since all other factors, including cellular tumor antigen levels and the *in vivo* growth rates of each tumor type, were similar. However, treatment with the apoptosis inducing nucleoside analogue gemcitabine was able to restore nuclear antigen cross-presentation to levels equivalent to secreted or cytoplasmic compartments. The antigen-dose dependent nature of this improvement suggested that treatment with gemcitabine boosted the amount of antigen available for cross-presentation and thus for cross priming. While the F1 cross-presentation model precludes any assessment of tumor regression due to the inability of direct antigen presentation, the increase in the proportion of CL4 CD8^+^ T cells associated with gemcitabine treatment and their ability to express IFNγ correlates to our earlier studies in which these CL4 cells drive an anti-tumour response against CL4 bearing tumours [25], [31]. It is therefore interesting to consider whether the reduced efficacy of nuclear tumor antigen cross-presentation before gemcitabine treatment might play a crucial role in tumor development, given that tumor antigen detection is critical for effective immune surveillance that protects the host during cancer development [34]. One possible interpretation is that the relatively reduced level of nuclear antigen cross-presentation might correlate with less effective immunosurveillance, limiting recognition of nuclear antigen bearing tumors by the host immune system. This hypothesis fits with the observation that cancer patients with tumor antigen localized in the nucleus have a more aggressive disease progression than patients with the same antigen expressed in the cytoplasm, or other tumor cell compartments [15]–[19]. Alternatively, it might be that after gemcitabine treatment, newly synthesized antigen may not localize correctly to the nucleus, resulting in increased cytosolic antigen that led to the observed increase in cross-presentation. Importantly, the enhanced expression of nuclear tumor antigen following gemcitabine treatment has been observed in human cancers [14], [35] and while the exact mechanism involved remains uncertain, it has been suggested that gemcitabine induced apoptosis promotes nuclear fragmentation [21] and such fragments may lead to the exposure of nuclear contents [23] and an increased availability of nuclear-bound tumor antigen for cross-presentation. Taken together, these data suggest that the relatively reduced cross-presentation of nuclear localized antigen can be improved by a gemcitabine-mediated, tumor apoptosis-induced, nuclear degradation and this increases the availability of these nuclear antigen for cross-presentation. Future studies will assess the key questions of antigen localization following gemcitabine treatment and also determine how effective other chemotherapy drugs are in ‘exposing’ nuclear tumor antigens to T cells.

### Harnessing nuclear antigens to improve cancer immunotherapies

Combination chemo-immunotherapy has recently been shown to be a powerful anti-cancer strategy, provided the right combination of chemotherapy drugs and immunotherapeutic agents are used [5], [36]. Therefore, the data from studies like ours have the potential to inform the planning and development of future clinical protocols. For example, since TAAs like survivin and MAGE-A10 are nuclear localized in a variety of cancer types [12], [13], chemotherapy that augments the cross presentation of these antigens might boost the efficacy of survivin or MAGE-A10 vaccines. Furthermore, given the crucial role that cross-presentation also plays in thymic development of tolerance to TAAs [37], the reduced cross-presentation of nuclear localized antigen relative to cytoplasmic and secretory antigen, may result in a peripheral T cell repertoire of nuclear-TAA reactive T cells that is less tolerant than those T cells reactive to antigen from other compartments. Thus nuclear TAAs might conceivably be better targets for cancer immunotherapy. In this regard, it may be feasible to use the increased availability of nuclear antigen after chemotherapy (Fig. 4A) to ‘turn tumors into their own vaccines’ (reviewed in [38]). This, in turn, has the potential to enhance newly emerging immunotherapies such as anti-CTLA-4/anti-PD-1/L1 immune checkpoint blockade [39], [40].

In summary, nuclear tumor antigen are relatively less well cross-presented to anti-tumor T cells than those from the cytoplasmic and secretory compartments, but this situation can be reversed by an apoptosis-inducing chemotherapy.
